# What Is Pneumo-Typhus?

**Published:** 1869-08

**Authors:** 


### What is Pneumo-Typhus?

Nothing is more common than for Typhus to become complicated with Pneumonia whose invasion is seldom indicated by any unusual dyspnœa, or expectoration, or by any of its common symptoms. This so-called Pneumonia is often the cause of a fatal termination of typhus. In the young and robust, it may be suspected from the occurrence of a sudden and marked increase of fever; but in old or much enfeebled patients it comes on, marked only by a sudden prostration of strength and loss of consciousness; the skin becomes harsh, the excretions fetid, the teeth and tongue covered with a fuliginous coating; while coma and trachial rattle announce the near approach of death.

Cancer is also apt to be followed by Pneumonia with some of the above symptoms. In 22 cases the upper lobes were chiefly affected; very old people seldom recover. The symptoms of meningitis complicating pneumonia are obscured, the patient is often delirious, but he comes comatose towards the end.

In five cases there was jaundice, three of which were fatal, but no lesion of liver was found.

Of nine cases in first stage, only one proved fatal, and in that pneumonia was double; of forty-six in the second stage, seventeen died; of six cases in third stage all proved fatal. The older the patient and the more feeble, or diseased originally, of course the more fatal the case proved.—*Ib.*

